# Interventions to improve sexual health among the homeless community: a realist review protocol

**DOI:** 10.12688/hrbopenres.14193.1

**Published:** 2025-07-23

**Authors:** Hau Nguyen, John Gilmore, Rikke Siersbaek, Chris Noone

**Affiliations:** 1 University of Galway, Galway, County Galway, Ireland; 2 University College Dublin, Dublin, Leinster, Ireland; 3 The University of Dublin Trinity College, Dublin, Leinster, Ireland

**Keywords:** Sexual health, Homelessness, Intervention, Realist review

## Abstract

**Background:**

People experiencing homelessness face profound sexual health inequities, driven by overlapping structural vulnerabilities including housing instability, stigma, limited healthcare access, and social exclusion. Interventions designed to improve sexual health outcomes within this population are diverse, ranging from peer-led education to nurse-led clinical models and structural supports such as housing. However, the effectiveness of these interventions depends on the content of the interventions as well as varying significantly across circumstances and subpopulations. A realist approach is useful for understanding how, for whom, and under what circumstances interventions produce intended (and unintended) outcomes.

**Methods:**

This review will follow Pawson’s six-step realist synthesis methodology. An initial programme theory has been developed through preliminary literature searches and will be refined iteratively as new evidence is gathered. Published and grey literature will be systematically searched and selected for their relevance to developing and testing Context–Mechanism–Outcome Configurations. Data extraction will focus on identifying key information which can contribute to understanding contextual factors, mechanisms, and outcomes across intervention types. The synthesis will develop, refine, and test theories that explain how sexual health interventions produce change in varied contexts. Patient and public involvement contributors will be consulted throughout the review to validate findings and support interpretation.

**Conclusion:**

This review will generate transferable insights into how sexual health interventions for people experiencing homelessness work across different contexts. By identifying mechanisms that spark outcomes, and the conditions that shape them, the findings will support more context-sensitive and effective policy, practice, and service design. The results will be disseminated through peer-reviewed publications, practitioner-facing summaries, and community-informed outputs.

## Introduction

Homelessness is a complex and dynamic condition that manifests in multiple forms. It is frequently classified into unsheltered homelessness (e.g., sleeping rough or in vehicles), sheltered homelessness (e.g., use of emergency accommodation), and hidden homelessness (e.g., temporary stays with friends or family, often unrecorded in official statistics). The European Typology of Homelessness and Housing Exclusion (ETHOS) offers a nuanced conceptual framework detailing the full spectrum of the experience of homelessness, encompassing rooflessness, houselessness, insecure housing, and inadequate housing ^1^. This typology is particularly relevant for research adopting a realist approach, as it supports an examination of how varying housing circumstances shape vulnerability and access to support. Across jurisdictions, definitions of homelessness vary based on policy, legal, and service delivery frameworks. In the U.S., homelessness is narrowly defined by the Department of Housing and Urban Development (HUD) based on the absence of a fixed, adequate nighttime residence ^2^. In contrast, Canada and Ireland adopt broader definitions. Ireland’s Housing Act of 1988, for instance, includes those whose accommodation is unsuitable or poses a risk to health and safety ^3^. These definitional differences reflect deeper structural and cultural assumptions about the causes and consequences of homelessness and inform how states respond.

As of early 2025, homelessness in Ireland has reached unprecedented levels, with 14,864 people recorded in emergency accommodation—the highest since national records began ^4^. Homelessness is underpinned by poverty, housing precarity, and systemic exclusion, and is associated with considerable disparities in physical and mental health ^5, 6^. Sexual health is a particularly neglected domain. The World Health Organization (WHO) defines sexual health as a state of physical, emotional, mental, and social well-being in relation to sexuality. It is not merely the absence of disease or dysfunction but requires a positive and respectful approach to sexuality and sexual relationships, as well as the possibility of having pleasurable and safe sexual experiences, free from coercion, discrimination, and violence ^7^.

Sexual health encompasses a wide range of issues, including access to contraception, prevention, and treatment of sexually transmitted infections (STIs), sexual education, and the ability to engage in consensual, safe sexual activities. For the people experiencing homelessness, maintaining sexual health is particularly challenging due to the instability and risks associated with their living situations. Research indicates that people experiencing homelessness are more likely than their housed counterparts to engage in high-risk sexual behaviours, such as having unprotected sex, engaging in transactional sex, or having multiple sexual partners ^8, 9^. These behaviours, combined with limited access to sexual health services, stigma, and the transient nature of homelessness contribute to elevated rates of STIs and other sexual health issues within this community ^10, 11^.

Existing reviews of sexual health interventions targeting people experiencing homelessness and other high-risk populations highlight promising strategies but suffer from several shared limitations in scope and methodology. Wright and Walker conducted a narrative systematic review of sexual health interventions among homeless people who use drugs ^12^. While they identified potentially effective behavioural and attitude change interventions, their findings were constrained by a limited six included studies, and a lack of exploration into how circumstantial and structural factors influenced outcomes and is now outdated. A systematic review by Naranbhai *et al.* focused specifically on HIV prevention among young people experiencing homelessness ^13^. The authors were only able to include three RCTs for their review. They concluded that the lack of quality evidence and the heterogeneity in intervention characteristics hindered the synthesis of evidence regarding the effectiveness of these interventions. Brown *et al.* reviewed behavioural and psychosocial interventions for youth in high-risk communities ^14^. Like Naranbhai *et al.*’s review, the authors only included three studies in their review with regards to the homeless community without being able to come to conclusions as to how these interventions might work or whether they are effective as the studies were too heterogeneous to synthesise.

Notably, the aforementioned reviews were mostly restricted to trials, either intentionally or unintentionally as most non-trial studies did not meet the quality criteria to be included, which limit their ability to account for the complexity of homelessness and healthcare interventions as well as their broader circumstances. Prioritising trials also risks leaving out broader implementation studies or qualitative evaluations that could provide richer insight into how interventions function the real world. Together, these reviews suggest that while there is emerging evidence of what works, the reliance on trials—though methodologically rigorous—often excludes valuable insights from mixed-methods, qualitative, or naturalistic studies. Such exclusions are particularly problematic in the homelessness field, where lack of funding, instability, and ethical concerns make controlled trials challenging. This highlights a pressing need for a different method for evidence synthesis that includes a broader evidence base and which is specifically designed to understand complex interventions and circumstances.

### Rationale

Homelessness is a significant public health issue, including in high-income nations such as Ireland ^15^. Communities experiencing homelessness face profound disparities in physical, mental, and sexual health due to structural and circumstantial factors, including housing instability, economic insecurity, and limited access to healthcare services. Sexual health is an often overlooked but critical aspect of wellbeing for people experiencing homelessness. Research highlights elevated rates of sexually transmitted infections (STIs), unintended pregnancies, and survival sex among this community ^15^. However, key questions remain unanswered: In what ways, for whom, and under what conditions do sexual health interventions work—or fail—by triggering certain mechanisms within particular settings and life situations?

A realist review is uniquely suited to address these questions. By examining a broader range of evidence—including but not limited to trials—this approach focuses on developing, refining, and testing programme theories that explain how latent powers (mechanisms) are triggered in a given context to produce outcomes ^16^. Rather than asking simply whether an intervention works, this review will explore how and why it works (or fails), for whom, and in what circumstances.

### The current review

This realist review aims to fill the gaps identified above by systematically synthesising the published evidence to explain how sexual health interventions for homeless populations work, for whom, and under what conditions. The review will identify causal patterns of change across intervention types (e.g., peer-led, nurse-led, educational, structural) and settings, and highlight how contextual factors (e.g., housing status, service delivery models) shape outcomes. Through the development of context–mechanism–outcome configurations (CMOCs) and theories, this realist review will provide policymakers, practitioners, and service planners with a deeper understanding of how to design and deliver more responsive, equitable, and sustainable sexual health interventions for people experiencing homelessness.

## Protocol

This realist review seeks to explore how and why sexual health interventions for people experiencing homelessness lead to different outcomes, depending on individual circumstances and broader social circumstances. This protocol is registered on PROSPERO (CRD420251047807).

## Methods

The current review will be conducted following a structured, theory-driven process composed of six iterative steps according to Pawson’s guidelines ^16^. These include: (i) identifying the review question, (ii) searching for primary studies, (iii) quality appraisal, (iv) extracting the data, (v) synthesizing the data and (vi) disseminating the findings.

First, the scope of the review is clarified by defining the research question in realist terms—focusing on what works, for whom, in what circumstances, and how—and by developing an initial programme theory to guide inquiry. Second, a systematic search for evidence is conducted across diverse sources, including both academic and grey literature, to identify data relevant to understanding how interventions function. Third, the evidence is appraised and extracted based on the relevance to the research question and the rigour of the study which produced it. Fourth, evidence is synthesised through the development and refinement of CMOCs, and then ultimately producing theories that explain how mechanisms are triggered under different contextual conditions to produce outcomes. Iterative data searching can take place at any point during the steps 2–4. Fifth, the programme theory is continually refined and tested throughout the review process as needed. Finally, the findings are disseminated in ways that are useful to policymakers, practitioners, and other stakeholders, emphasising transferable insights about how interventions operate.

### Initial programme theory

Through a preliminary informal review of the literature, the authors identified a range of sexual health interventions targeting people experiencing homelessness, most of which can be categorised as one of the following categories: nurse-led, peer-led, educational or structural interventions. These interventions, varied in their design, delivery agents, and assumptions about how change occurs. Further synthesis revealed that these approaches often share underlying mechanisms and interact in complementary ways. Consequently, the authors developed an initial programme theory that captures the common mechanisms, contextual influences, and outcome pathways across these diverse interventions. This initial theory was subsequently refined through consultation with an expert advisory group, composed of professionals with experience in sexual health, homelessness, and social inclusion. Feedback gathered during a facilitated advisory panel meeting helped ensure the theory’s relevance, coherence, and grounding in real-world practice and research. Details of the initial programme theory can be found at the Open Science Framework (OSF): Interventions to improve sexual health among the homeless community: a realist review protocol.

https://doi.org/10.17605/OSF.IO/W9VXY ^17^.

### Searches

Following the principles outlined by Pawson ^16^, the literature search for this realist review has been conducted in two phases. An initial, exploratory search was undertaken to identify key intervention types, contextual influences, and candidate mechanisms relevant to promoting sexual health among people experiencing homelessness. Along with the team’s subject area knowledge, this pilot search informed the development of an initial programme theory, which will guide the selection of search terms. A second systematic search will be undertaken with the assistance of a subject librarian. The search strategy used at the initial programme theory development stage can be found at the Open Science Framework (OSF): Interventions to improve sexual health among the homeless community: a realist review protocol.

https://doi.org/10.17605/OSF.IO/W9VXY ^17^.

### Inclusion and exclusion criteria

This review will include studies of any design—quantitative, qualitative, or mixed-methods—that provide data relevant to understanding how sexual health interventions work for people experiencing homelessness. Eligible studies must contain information that can contribute to the development or refinement of CMOCs, regardless of whether they report on intervention effectiveness. The main databases to be searched are CINAHL - Cumulative Index to Nursing and Allied Health Literature, Embase.com, MEDLINE and PsycINFO. Grey literature, policy documents, and theoretical papers will also be included where they offer explanatory insight. Studies that are not published in English will not be included. PICO criteria are as followed:

*Population:*

Included people experiencing homelessness or unstable housing, including subgroups (e.g., youth, LGBTQ+, those with mental health needs or substance use)

*Intervention(s) or exposure(s):*

Included sexual health interventions of any type (e.g., peer-led, nurse-led, educational, structural), including those integrated into wider health or social services

*Comparator(s) or control(s):*

This review does not have any comparators

*Study design:*

Both randomized and nonrandomized study types will be included.

### Data extraction and synthesis

Covidence will be used to screen retrieved articles. Titles and abstracts will be double screened to ensure consistency. Discrepancies will be discussed, and if consensus cannot be reached, a third reviewer will be consulted to make the final decision. The same process will be applied during full-text screening.

***Expert advisory group.*** This review includes structured involvement from an expert advisory group, in line with best practice principles for ethical and meaningful engagement as outlined by the Irish Health Research Forum ^18^. This review adopts a consultation-based approach whereby experts’ consultation will be sought twice- at the initial programme theory development stage and the study completion stage. The group is composed of individuals with expertise in frontline service provision, sexual health, social inclusion, and homelessness policy. The panel includes representatives from the Health Service Executive (HSE), Safetynet Primary Care, and the Dublin Simon Community—all of whom are directly involved in delivering or coordinating services for people experiencing homelessness. It also includes academics from Trinity College Dublin, University of Galway, and University College Dublin, whose research engages directly or indirectly with issues related to homelessness, health equity, and vulnerable populations. Together, the group brings a well-rounded and practice-informed perspective to the development and refinement of the review’s programme theory.

In the first phase, the group met to discuss and shape the development of the initial programme theory. This session was facilitated to gather expert insights and test the early conceptual thinking behind how sexual health services may or may not work for people experiencing homelessness. The discussions helped identify key contextual factors, mechanisms, and outcomes to guide the subsequent literature review. A second consultation will take place once the realist synthesis is complete. At that stage, the group will be invited to provide feedback on the refined, overarching programme theory, offering critical reflections on its coherence, resonance with real-world practice, and potential relevance to policy and service design. We aim to include expertise through lived experience at this stage.

## Dissemination

Results will be shared via peer-reviewed journal publications and conference presentations in the fields of public health, homelessness, and sexual health. In alignment with our commitment to patient and public involvement, accessible summaries will also be co-developed with public and patient involvement (PPI) contributors to share findings in plain language through community organisations, outreach networks, and advocacy groups. Key insights—particularly refined CMOCs—will be presented in visual formats such as logic models or evidence maps to support policy and practice uptake without the use of research jargon. Additionally, findings will be shared with stakeholders in health and homelessness services to inform future intervention design, delivery, and commissioning.

## Conclusion

This realist review protocol outlines a comprehensive plan to investigate how sexual health interventions function for people experiencing homelessness, for whom, and under what circumstances. Moving beyond traditional systematic reviews which are designed to synthesise trial-based outcomes and effect sizes, this approach will synthesise evidence from a diverse range of study designs. By developing and testing CMOCs, the review aims to build theories that are transferable across settings. This theory-driven synthesis will help uncover the underlying mechanisms that shape intervention success or failure, while accounting for key contextual factors such as housing status and access to care. Meaningful patient and public involvement (PPI) will guide the review to ensure its relevance and impact. Ultimately, this work will inform more equitable, effective, and sustainable approaches to sexual health for the homeless community.

## Review stage

The review has completed the IPT development stage, where the IPT was brought in for discussion with the expert advisory group to gather their insights and feedback. The next phase of the review will involve an in-depth literature review and realist data synthesis, aimed at testing and further refining the programme theories.

## Ethical considerations

There are no ethical considerations necessary as this is a review study synthesising published literature. No primary data will be collected.

## Data Availability

No data associated with this article. Open Science Framework (OSF): Interventions to improve sexual health among the homeless community: a realist review protocol, https://doi.org/10.17605/OSF.IO/W9VXY ^17^. This project contains the following underlying data: PRISMA-P checklist, Search strategies, Initial programme theory. Data are available under *CC-By Attribution 4.0 International license.*
