# Supplementary material for: Interventions to improve sexual health among the homeless community: a realist review protocol
Source: HRB Open Res. 2026 May 5;8:83. Originally published 2025 Jul 23. [Version 2] doi: 10.12688/hrbopenres.14193.2 (PMC13191315; doi:10.12688/hrbopenres.14193.2)
Supplement: Peer review history

# Reviewer report: Reviewer response for version 2

**DOI:** 10.21956/hrbopenres.15906.r55199

**Published:** 2026-05-23

**Authors:** Jessica Sherman

**Affiliations:** 1 The Ohio State University, Columbus, Ohio, USA

Thank you for the opportunity to review this protocol. I have reviewed the manuscript alongside the prior review and the authors’ responses. The protocol is clear, concise, and well written. The authors provide both a thorough description of the methods and a strong justification for their approach. They have also responded thoughtfully to prior reviewer comments, and the manuscript is strengthened as a result.

 The Introduction is clear and well developed. The authors effectively convey the complexity of homelessness, including variations in definitions across countries, and provide a succinct and appropriate synthesis of the existing literature.

 The Methods are well described, with sufficient detail to ensure clarity for readers who may be unfamiliar with realist methods. The authors also provide a strong rationale for using this approach to examine the sexual health of people experiencing homelessness.

 Overall, this is a well-written manuscript that applies a realist approach to an important topic. It has strong potential to advance understanding of what works (and what does not) in efforts to support the sexual health of people experiencing homelessness.

Is the study design appropriate for the research question?

Yes

Is the rationale for, and objectives of, the study clearly described?

Yes

Are sufficient details of the methods provided to allow replication by others?

Yes

Are the datasets clearly presented in a useable and accessible format?

Not applicable

Reviewer Expertise:

homelessness, sexual health, HIV, LGBGT+ health, mental health, substance use

I confirm that I have read this submission and believe that I have an appropriate level of expertise to confirm that it is of an acceptable scientific standard.

# Reviewer report: Reviewer response for version 2

**DOI:** 10.21956/hrbopenres.15906.r55116

**Published:** 2026-05-20

**Authors:** Martha Paisi

**Affiliations:** 1 University of Plymouth, Plymouth, UK

Thank you for your email and patience.

 I have revised the newer version and I believe in its current form, it is suitable for indexing.

Is the study design appropriate for the research question?

Yes

Is the rationale for, and objectives of, the study clearly described?

Yes

Are sufficient details of the methods provided to allow replication by others?

Yes

Are the datasets clearly presented in a useable and accessible format?

Yes

Reviewer Expertise:

NA

I confirm that I have read this submission and believe that I have an appropriate level of expertise to confirm that it is of an acceptable scientific standard.

# Reviewer report: Reviewer response for version 2

**DOI:** 10.21956/hrbopenres.15906.r55115

**Published:** 2026-05-09

**Authors:** Wendy Masterton

**Affiliations:** 1 University of Stirling, Stirling, UK

I think the authors have done a good job of addressing my comments and have edited the article accordingly. The additions in methods are particularly helpful for clarity relating to the methodological approach and the CMOCs/realist terminology. The additional considerations around the advisory groups and dissemination also strengthens the protocol. I look forward to reading the review in the future.

Is the study design appropriate for the research question?

Yes

Is the rationale for, and objectives of, the study clearly described?

Yes

Are sufficient details of the methods provided to allow replication by others?

Partly

Are the datasets clearly presented in a useable and accessible format?

Not applicable

Reviewer Expertise:

substance use; harm reduction; mental health; realist methodology

I confirm that I have read this submission and believe that I have an appropriate level of expertise to confirm that it is of an acceptable scientific standard.

# Reviewer report: Reviewer response for version 1

**DOI:** 10.21956/hrbopenres.15609.r53416

**Published:** 2026-02-26

**Authors:** Wendy Masterton

**Affiliations:** 1 University of Stirling, Stirling, UK

Thank you for the opportunity to review this protocol. It is an important topic, the protocol is considered and well written, and I am convinced by the realist approach. I have a few comments/suggestions that may strengthen it moving forward.

**Introduction**

 I am glad to see the complexity of the homelessness definition discussed as this is important.

 I did find myself wondering what the geographical focus of the review was. The authors helpfully discuss the comparisons around homelessness definitions internationally, but the focus on Ireland becomes more apparent from paragraph 2. If the review is specific to Ireland and informing Irish policy/practice, I think this needs to be made explicit in the abstract, if not the title, as well as the aim of the current review. If it is designed to be internationally relevant, I think there needs to be a little bit of editing in the Introduction section to ensure it doesn’t read as Ireland-focused.

 Otherwise, the general evidence gap around the topic was clear to me, so with the above clarity, the rationale is strong.

 The authors shows good knowledge of realist methodology, but the introduction would benefit from some definition work around realist terms. The term ‘programme theory’ is only loosely defined, and it isn’t clear (with a realist lens) what context means. The authors may also want to add a little about context-mechanism-outcome configurations (CMOcs) as this is more implied than explicit. Realist terminology is notoriously jargony, and clarity is essential.

**Methods**

 The review will be clearly mapped against Pawson’s guidelines which is good. It would further strengthen the methods to state that the review will be conducted and reported in line with the RAMESES realist review standards too as I would expect these to be followed/mentioned: [Ref 1]

 Helpful information provided about the IPT – this is so often missed/glossed over, so I applaud the authors for clearly explaining the IPT process and the importance of this.

 Extensive search strings which is also good to see, particularly given the complexity of the topic and many different potential terms used.

 It might be helpful to really emphasise that any and all types of studies/resources can be used in a realist review, this is one of the key differences from systematic reviews and is important if there is evidence in unpublished work (which I imagine there will be given the topic and organisational reports etc).

 I do always question why authors decide to not include studies that are not in English without any rationale provided. There are pretty accurate free translation online now that translates articles almost immediately and to a high quality. I don’t see what the rationale is for excluding these? The exclusion may be linked to relevance to Irish context? If so, make this relevance-related consideration clearer.

 There could be more clarity around synthesis. How will data be synthesised and tested against the IPTs? Will it be via NVivo or by hand?

 Inclusion of the advisory group is good, and the detail around how and when they contributed is clear. However, in the paper itself, the authors will need to provide more detail around the lived experience inclusion. Realist work can be quite difficult to gain consultation on, particularly with groups who haven’t worked in a research field before, so consideration of this, and how it will be reported, will be essential. This will also be relevant when thinking about dissemination and how this could look for non-academic audiences. This is briefly mentioned in the protocol, so I would urge the authors to give ample time to this.

Is the study design appropriate for the research question?

Yes

Is the rationale for, and objectives of, the study clearly described?

Yes

Are sufficient details of the methods provided to allow replication by others?

Partly

Are the datasets clearly presented in a useable and accessible format?

Not applicable

Reviewer Expertise:

substance use; harm reduction; mental health; realist methodology

I confirm that I have read this submission and believe that I have an appropriate level of expertise to confirm that it is of an acceptable scientific standard, however I have significant reservations, as outlined above.

## References

[1] : RAMESES publication standards: realist syntheses. *BMC Medicine* .2013;11(1) : 10.1186/1741-7015-11-21 10.1186/1741-7015-11-21 23360677 PMC3558331

# Response

**Published:** 2026-04-15

**Authors:** Hau Nguyen

**Affiliations:** School of Psychology, University of Galway, Galway, County Galway, Ireland

**Masterton’s Comment Address**

**1.** **Introduction**

 I am glad to see the complexity of the homelessness definition discussed as this is important.

 I did find myself wondering what the geographical focus of the review was. The authors helpfully discuss the comparisons around homelessness definitions internationally, but the focus on Ireland becomes more apparent from paragraph 2. If the review is specific to Ireland and informing Irish policy/practice, I think this needs to be made explicit in the abstract, if not the title, as well as the aim of the current review. If it is designed to be internationally relevant, I think there needs to be a little bit of editing in the Introduction section to ensure it doesn’t read as Ireland-focused.

 Otherwise, the general evidence gap around the topic was clear to me, so with the above clarity, the rationale is strong.The authors shows good knowledge of realist methodology, but the introduction would benefit from some definition work around realist terms. The term ‘programme theory’ is only loosely defined, and it isn’t clear (with a realist lens) what context means. The authors may also want to add a little about context-mechanism-outcome configurations (CMOcs) as this is more implied than explicit. Realist terminology is notoriously jargony, and clarity is essential.

**Answer:**

 Thank you for this helpful observation. The review aims to synthesise international evidence on sexual health interventions for people experiencing homelessness in order to develop programme theories that may be transferable across contexts. While the growing homelessness crisis in Ireland partly motivates the study, the review itself is not restricted to Irish evidence. We have revised the introduction by adding statistics from several other countries ensure the text does not appear overly Ireland-focused.

 Regarding the comment regarding the programme theory, we believe that it is in regard of the method section because that is where the term was first introduced. The method section hence has been revised to provide clearer definitions of key realist concepts, including programme theory, context, and context–mechanism–outcome configurations (CMOCs), to improve accessibility for readers unfamiliar with realist methodology.

**2. Methods**

 The review will be clearly mapped against Pawson’s guidelines which is good. It would further strengthen the methods to state that the review will be conducted and reported in line with the RAMESES realist review standards too as I would expect these to be followed/mentioned: [Ref 1]

 Helpful information provided about the IPT – this is so often missed/glossed over, so I applaud the authors for clearly explaining the IPT process and the importance of this.

 Extensive search strings which is also good to see, particularly given the complexity of the topic and many different potential terms used.

 It might be helpful to really emphasise that any and all types of studies/resources can be used in a realist review, this is one of the key differences from systematic reviews and is important if there is evidence in unpublished work (which I imagine there will be given the topic and organisational reports etc).

 I do always question why authors decide to not include studies that are not in English without any rationale provided. There are pretty accurate free translation online now that translates articles almost immediately and to a high quality. I don’t see what the rationale is for excluding these? The exclusion may be linked to relevance to Irish context? If so, make this relevance-related consideration clearer.

**Answer:**

 Thank you for your helpful suggestions. We have reported that the review will be conducted and reported in line with the RAMESES realist review standards **. **We have reflected that any and all types of studies/resources can be used in a realist review in the method section. We have also clarified that qualitative data management software NVivo will be used to support coding and organisation of data during the synthesis process.

 While we acknowledge that machine translation tools have improved considerably, the interpretation of qualitative and contextual information remains particularly important in realist reviews, where nuanced descriptions of mechanisms, context, and programme implementation are central to theory development. Automated translation may not reliably capture these subtleties, which could affect the interpretation of context–mechanism–outcome relationships. In addition, the review team does not have the resources to conduct formal verification of machine-translated texts. For these reasons, the review will be limited to studies published in English.

**3. Data extraction and synthesis**

 There could be more clarity around synthesis. How will data be synthesised and tested against the IPTs? Will it be via NVivo or by hand?

**Answer:**

 Thank you for this important comment. We have expanded the Methods section to provide greater clarity on how data will be synthesised and tested against the initial programme theory (IPT). Data synthesis will follow realist principles, involving iterative comparison of extracted data with the IPT to refine, confirm, or refute emerging context–mechanism–outcome configurations (CMOCs). This process will involve identifying patterns across studies, developing and testing explanatory propositions, and refining programme theories accordingly. We have also clarified that qualitative data management software (e.g., NVivo) may be used to support coding and organisation of data, although the analytic process will remain theory-driven and iterative rather than dependent on a specific software tool.

**4.Expert Advisory Panel and Dissemination**

 Inclusion of the advisory group is good, and the detail around how and when they contributed is clear. However, in the paper itself, the authors will need to provide more detail around the lived experience inclusion. Realist work can be quite difficult to gain consultation on, particularly with groups who haven’t worked in a research field before, so consideration of this, and how it will be reported, will be essential. This will also be relevant when thinking about dissemination and how this could look for non-academic audiences. This is briefly mentioned in the protocol, so I would urge the authors to give ample time to this.

**Answer: **Thank you for this important observation. We recognise that realist methodology can be conceptually complex and that meaningful engagement with individuals who may not have prior research experience requires careful consideration. The protocol has been revised to provide further clarification on how individuals with lived experience of homelessness will be supported to engage with the review process. Lastly, the dissemination section has been revised to clarify that outputs will be developed in accessible formats and shared through community organisations and outreach networks to ensure that findings are accessible not only to practitioners and policymakers but also to people experiencing homelessness themselves.

# Reviewer report: Reviewer response for version 1

**DOI:** 10.21956/hrbopenres.15609.r48576

**Published:** 2025-08-22

**Authors:** Martha Paisi

**Affiliations:** 1 University of Plymouth, Plymouth, UK

Thank you for the opportunity to review this manuscript. This is a well-articulated and methodologically sound realist review protocol that seeks to explore how, why, and under what circumstances sexual health interventions work for people experiencing homelessness. The protocol is timely, relevant, and aligned with a growing need to understand complex health interventions in vulnerable populations.

 It adheres closely to established realist methodology and shows a clear commitment to rigour, stakeholder involvement, and practical impact. The topic addresses a significant gap in the literature by moving beyond effectiveness and toward contextual understanding and theory building. I would recommend 'Accept with minor revisions'.

**1.Abstract**

**Strengths**:

- The abstract succinctly captures the aims, rationale, and methodological approach.

**Recommendations:**

- The complexity of lived experience could be introduced in the background section (it is appropriately discussed in detail later in the main text).
- Consider briefly defining “CMOC” or “realist review” for broader accessibility.

**2. Introduction**

**Strengths:**

- Provides strong contextual background with international framing (e.g., ETHOS typology).
- The complexity of both the definition and lived experience of homelessness is well captured.
- Articulates the relevance of sexual health as a neglected domain among people experiencing homelessness.
- Well-referenced and connects with policy and public health literature.

**Recommendations:**

- Slight repetition of critique regarding previous reviews may be condensed.

**3. Rationale and Objectives**

**Strengths:**

- Strong justification for adopting a realist approach.
- Clearly identifies the gaps in the evidence base regarding sexual health interventions.

**4. Methods**

**Strengths:**

- Adheres well to established realist methodology.
- Describes each stage of the synthesis clearly.
- Strong emphasis on theory development, iteration, and stakeholder input.

**Recommendations:**

- Clarify how rigour and relevance will be assessed in included studies.
- Consider elaborating on whether additional software (e.g., NVivo) will be used for qualitative synthesis.
- Provide a short example or visualisation of how a CMOC might be constructed.

**5. Programme Theory Development**

**Strengths:**

- The use of an expert advisory group enhances credibility and grounding in practice.
- Transparency is ensured via registration on OSF and PROSPERO.

**Recommendations:**

- Consider summarising or visualising the initial programme theory within the protocol.

**6. Search Strategy and Inclusion Criteria**

**Strengths**:

- Inclusive of a broad range of study types and grey literature aligned with realist principles.
- Appropriate databases selected.
- Clear inclusion criteria using PICO-like structure adapted for realist review.

**Recommendations**:

- To clarify whether the reference lists of included papers will be examined.

**7. Data Extraction and Synthesis**

**Strengths**:

- Plans for double screening and conflict resolution are clearly stated.
- Emphasis on CMOCs reflects strong realist methodology.

**Recommendations**:

- Further clarification on how different types of data (qualitative vs quantitative) will be integrated into CMOCs would strengthen the section.
- A worked example of a CMOC could aid reader understanding.

**8. Stakeholder and PPI Engagement**

**Strengths**:

- Strong and ethical engagement strategy, including frontline providers and policymakers.
- Intention to include individuals with lived experience at later stages is commendable and would strongly recommend.

**Recommendations**:

- Consider more concrete description of how PPI will shape dissemination or interpretation of findings.

**9.  Dissemination**

**Strengths**:

- Good dissemination plans targeting academic and practitioner audiences.
- Use of visual summaries and non-academic formats is excellent.

**Recommendations**:

- Could consider discussing how findings might be made accessible to people experiencing homelessness themselves.

Is the study design appropriate for the research question?

Yes

Is the rationale for, and objectives of, the study clearly described?

Yes

Are sufficient details of the methods provided to allow replication by others?

Yes

Are the datasets clearly presented in a useable and accessible format?

Yes

Reviewer Expertise:

Health equity, access to services for people expericning homelessness

I confirm that I have read this submission and believe that I have an appropriate level of expertise to confirm that it is of an acceptable scientific standard, however I have significant reservations, as outlined above.

# Response

**Published:** 2026-04-15

**Authors:** Hau Nguyen

**Affiliations:** School of Psychology, University of Galway, Galway, County Galway, Ireland

**Paisi’s Comment Address**

**1.** **Abstract:**

 Strengths:

- The abstract succinctly captures the aims, rationale, and methodological approach.

Recommendations:

- The complexity of lived experience could be introduced in the background section (it is appropriately discussed in detail later in the main text).
- Consider briefly defining “CMOC” or “realist review” for broader accessibility.

**Answer: **Thank you for this helpful suggestion. We have revised the background section of the abstract to acknowledge the complex lived experiences of people experiencing homelessness. In addition, we have included a brief clarification of realist review terminology and context–mechanism–outcome configurations (CMOCs) to improve accessibility for readers unfamiliar with realist methodology.

**2.Rationale and Objective**

 Strengths:

- Strong justification for adopting a realist approach.
- Clearly identifies the gaps in the evidence base regarding sexual health interventions  

**3. Methods**

 Strengths:

- Adheres well to established realist methodology.
- Describes each stage of the synthesis clearly.
- Strong emphasis on theory development, iteration, and stakeholder input.

Recommendations:

- Clarify how rigour and relevance will be assessed in included studies.
- Consider elaborating on whether additional software (e.g., NVivo) will be used for qualitative synthesis.
- Provide a short example or visualisation of how a CMOC might be constructed.

**Answer:** Thank you for this helpful suggestion. The methods section has been expanded to provide a clearer explanation of how rigour will be assessed.

 An illustrative example of a context–mechanism–outcome configuration (CMOC) has been added to the Methods section to help readers unfamiliar with realist methodology understand how CMOCs will be constructed during synthesis.

 We have also clarified that qualitative data management software NVivo will be used to support coding and organisation of data during the synthesis process.

**4.Initial Programme Theory**

 Programme Theory Development

 Strengths:

- The use of an expert advisory group enhances credibility and grounding in practice.
- Transparency is ensured via registration on OSF and PROSPERO.

Recommendations:

- Consider summarising or visualising the initial programme theory within the protocol.

**Answer: **Thank you very much for your suggestions. We have now visualise the initial programme theory within the protocol

**5.Search Strategy and Inclusion Criteria**

 Strengths:

- Inclusive of a broad range of study types and grey literature aligned with realist principles.
- Appropriate databases selected.
- Clear inclusion criteria using PICO-like structure adapted for realist review.

Recommendations:

- To clarify whether the reference lists of included papers will be examined.

**Answer**: Thank you for your suggestion to clarify whether we will conduct backward citation for our searches. We can confirm that we do, and the following line has now been added to the section: “In addition, the reference lists of included studies will be examined to identify additional relevant sources that may not have been captured in the database searches.”

**6.Data extraction and synthesis**

 Strengths:

- Plans for double screening and conflict resolution are clearly stated.
- Emphasis on CMOCs reflects strong realist methodology.

Recommendations:

- Further clarification on how different types of data (qualitative vs quantitative) will be integrated into CMOCs would strengthen the section.
- A worked example of a CMOC could aid reader understanding.

**Answer**: Thank you for your recommendation, we did add an example in section 4 of the manuscript.

**7.Dissemination**

 Strengths:

- Good dissemination plans targeting academic and practitioner audiences.
- Use of visual summaries and non-academic formats is excellent.

Recommendations:

- Could consider discussing how findings might be made accessible to people experiencing homelessness themselves.

**Answer: **Thank you for this suggestion. The dissemination section has been revised to clarify that outputs will be developed in accessible formats and shared through community organisations and outreach networks to ensure that findings are accessible not only to practitioners and policymakers but also to people experiencing homelessness themselves.
